# Health Education to Reduce Helminthiasis: Deficits in Diets in Children and Achievement of Students of Elementary Schools at Pontianak, West Kalimantan

**DOI:** 10.1155/2020/4846102

**Published:** 2020-07-21

**Authors:** Widyana Lakshmi Puspita, Khayan Khayan, Didik Hariyadi, Taufik Anwar, Slamet Wardoyo, Bagus Muhammad Ihsan

**Affiliations:** ^1^Department of Nutrition, Poltekkes Kemenkes Pontianak, Pontianak, Indonesia; ^2^Department of Environmental Health, Poltekkes Kemenkes Pontianak, Pontianak, Indonesia; ^3^Post Graduate of Department of Parasitology and Microbiology, Medicine Faculty, Universitas Padjadjaran, Bandung, Indonesia; ^4^Department of Medical Laboratory Technology, Poltekkes Kemenkes Banten, Serang, Indonesia

## Abstract

Worms are still a serious problem for poor and developing countries. Children, especially school-age children, are more at risk of infection. Efforts need to be made to prevent the effects of worms. Prevention can be done through a promotive approach. This observational study with a pre-posttest and cross-sectional approach is aimed at evaluating the effectiveness of health education on healthy and helminthic behavior and analyzing the impact of helminthiasis on the deficit in the diet and children's learning achievement. The number of samples is 60 students from five grade 3 and 4 elementary schools in North Pontianak, West Kalimantan. The sampling technique was carried out by proportional random sampling. Worm infection in elementary school students was 16.7%, anemia was 55%, and learning achievement scores were less than the average grade of 55%. There was a significant difference in health counseling towards a decrease in the worm number (*p* = 0.046). There was a significant relationship between healthy living behavior and helminthiasis (*p* = 0.005). There was a significant relationship between helminthiasis and anemia (*p* = 0.017). There is a relationship between helminthiasis and learning achievement in elementary school children (*p* = 0.017). There is a relationship between anemia and learning achievement (*p* = 0.005). It is necessary for public health centers to provide treatment services for worms and health education about the effects of helminthiasis on health and learning achievement. The school should provide hand washing facilities in schools, and parents should play an active role in improving clean and healthy lifestyle habits at home.

## 1. Introduction

Public health service efforts, including those carried out through health education, should include nutritional status improvement and disease control [1]. Disease control in Indonesia experiences an epidemiological transition. On the hand, there is an increase in noncommunicable diseases such as hypertension and diabetes mellitus and in workplace accidents. On the other hand, there have not been any infections such as malaria, dengue fever, and worms [2].

Health problems caused by helminthiasis are still a public health problem [3, 4]. The WHO predicts the prevalence of helminthiasis to reach 2 billion people in the world, and in the case of children, the prevalence is expected to reach 61,300,000. In India, the prevalence in school-aged children is between 12.5 and 66% [4, 5]. In Indonesia, after a stool examination survey in elementary school children, helminthiasis was found to have a prevalence of 25%, and in Semarang, it was found to have the same prevalence of 25% [5, 6], whereas in North Sumatra, worm infection in children aged 1-5 years is 34.4% [7].

The types of worms that are found to contaminate elementary school children are mostly *Ascaris lumbricoides*, *Ancylostoma duodenale*, and *Trichuris trichiura* [4–7]. The high prevalence of helminthiasis in school-aged children is due to a lack of personal hygiene in elementary school children, poor personal health habits, such as doing activities that are more related to land, not wearing footwear while playing, and not washing hands and nails, and the poor environment of home and school sanitation [2, 7, 8].

Symptoms of mild helminthiasis in children are nausea, diarrhea, and reduced appetite. Children who are heavily infected can experience impaired food absorption (malnutrition), lack of enthusiasm, and reduced concentration in learning. Children infected with *Ascaris lumbricoides* worms will show characteristics such as a distended abdomen, pale eyes, frequent stomach pain, and reduced appetite [9]. Long-term helminthiasis in school-age children will cause disturbances, such as pain, laziness to go to school (absenteeism), and reduced nutritional status [5, 9]. Malnutrition in children will cause lethargy, anemia, and lack of enthusiasm for learning [10]. In severe and chronic infections causing children to experience malnutrition, physical growth and cognitive development can be interfered with [11]. Children will be intellectually disturbed, because of lack of nutrient intake (malnutrition), characterized by a decrease in the ability to learn and process new information, which results in children having difficulty developing their thinking power [10, 11].

Malnutrition can occur in children who have worm infections because the nutrients needed by the body are absorbed by the worms so that children experience nutritional anemia [11]. The incidence of nutritional anemia, due to deficiency in iron, folic acid, and/or vitamin B12, is rooted in inadequate nutrient intake, low bioavailability, and still high helminthiasis [11–13]. The impact of anemia, especially in school-age children, will result in paleness, fatigue, weakness, and decrease in antibody and reduced ability or concentration in learning. Children with anemia have a lower index of psychomotor development than healthy children [14]. Generally, their school performance is lower than that of normal children [15, 16].

At present, helminthiasis control based on elementary school children is still limited and rare. Government control, which is carried out by local community health centers, is limited to using a treatment approach. For this reason, efforts with a promotive approach to health and preventive counseling by improving clean and healthy living behaviors, as well as knowing the effects of helminthiasis such as anemia and poor learning achievement, can be known to reduce helminthiasis in elementary school children.

## 2. Materials and Methods

### 2.1. Research Design

This type of research is observational with a pre-posttest and cross-sectional approach. The aim of the research is to look at the description of health education variables, clean and healthy behavior, helminthiasis, and a deficit in diet related to the condition of anemia in children and learning achievement and then to analyze the relationship between these variables. This research was conducted on grade III and IV elementary school children in North Pontianak.

### 2.2. Subjects and Number of Samples

**Figure 1:**
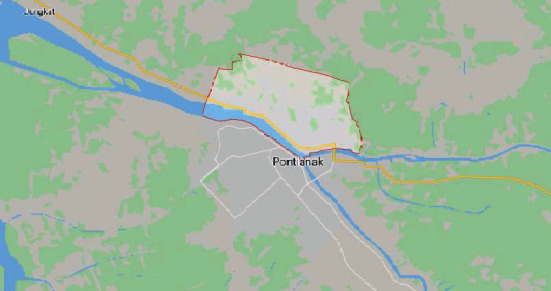
Research location map.

The research sample was taken from grade III and IV students from State Elementary Schools 01, 09, 32, and 33 and Hidayatullah Elementary School, in Siantan Hulu Village, North Pontianak, West Kalimantan (Figure 1). The sampling of elementary school students is based on the combination of the location of primary schools in urban areas (01 and 09) and elementary schools located on the outskirts of the city (32, 33, and Hidayatullah elementary schools). In accordance with the statistical formula, the number of samples is 60 students.

### 2.3. Sampling

The method of selecting samples is done by proportional random sampling for five primary schools in North Pontianak. The distribution of each elementary school is based on the number of students in grades III and IV. The number of samples for each elementary school is 15 students for 01 public elementary school, 6 students for 09 public elementary school, 11 students for 32 public elementary school, 16 students for 33 public elementary school, and 12 students for Hidayatullah elementary school. The sampling of students in grades III and IV of each primary school is done by a simple random sampling technique.

### 2.4. Research Instrument

The instruments used in this study are centrifuges for fecal examination (worms), Hb meters for blood measurement or examination (anemia), and grade III and IV report cards collected by tracking school documents for learning achievement data and characteristics of schoolchildren. Data on clean and healthy behavior is determined by observation with a checklist. There are two types of data in the study, namely, primary and secondary data. Primary data is taken directly by researchers in elementary schools, including data on worms and anemia. Secondary data include semester learning achievement data and characteristics of elementary school children, taken from documents held by elementary school students.

### 2.5. Data Collection

The data collected included fecal retrieval and examination (helminthiasis), implementation of health education, assessment of clean and healthy behavior, blood tests (anemia), and learning achievement. Data collection research was conducted in 3 stages. In the first stage, perform an examination of feces (worms) and blood hemoglobin, evaluate the value of learning achievement, and assess clean and healthy living habits. The second stage is the implementation of health education for elementary school children (grades III and IV). After the following week, health counseling was carried out again for the second time. In the third stage, after one month of intervention, the reexamination of feces (worms) and assessment of clean and healthy living behavior was carried out for students in grades III and IV and data on student achievement was collected. The research was conducted from January to August 2016.

#### 2.5.1. Fecal Examination

Study participants were given a clean labeled stool container containing 10 mL of 10% formalin. Toilet paper and clean sticks were given to collect the morning feces specimen the following day. Each child is instructed to bring their feces so that mixing does not occur. After collecting the stool specimens, they are immediately processed using a simple smear and the Kato-Katz thick smear technique. Sellotape slides were observed directly under a microscope for helminth eggs; then, identification was carried out. Hookworm, *Trichuris trichiura*, and *Ascaris lumbricoides* eggs were counted. The 10% subsample is used for quality control.

#### 2.5.2. Blood Tests (Identification of Anemia)

Hemoglobin examination is carried out by the cyanmethemoglobin method. A 10 *μ*L blood sample is put into a 2.5 mL bottle of Drapkin solution (a chemical or substance used for chemical analysis or synthesis), using a pipette, and then put into a serology tube, followed by gentle shaking until smooth; the solution is left for 3-5 minutes at room temperature; the photometer is read at a wavelength of 546 nm; and the results printed on the device screen are observed.

### 2.6. Data Analysis

Statistical analysis was performed using the Mann-Whitney proportional difference test used to analyze the effectiveness between before and after health counseling with a decrease in helminthiasis. The chi-square test was conducted to see the relationship between clean and healthy behavior and helminthiasis, analyze the link between helminthiasis and anemia, and evaluate the relationship between helminthiasis and anemia with learning achievement, with *α* = 5%.

### 2.7. Official Permission, Ethical Clearance, and Informed Consent

The study protocol was reviewed by the Health Education Ethics committee and given ethical permission. Official permission was obtained from the ethics committee Poltekkes Kemenkes Pontianak in West Kalimantan with the number 017/KEPK-PK.PKP/I/2016. Written approval was obtained from all parents of students and the school and Pontianak City Health Service.

## 3. Results

Anemia is a disease that is caused by lack of iron, folic acid, and vitamin B12 in the body which causes the formation of reduced hemoglobin. Iron deficiency anemia is also affected by the consequences of helminthiasis with chronic blood loss. From Table 1, it can be seen that 63.3% are mostly males. 16.7% of the respondents experienced helminthiasis, most respondents (55%) had anemia, the value of learning achievement was mostly below average (55%), and the clean and healthy life behavior is mostly good (51.7%). With regard to the education of parents of students, 50% of the mothers and 41.7% of the fathers had primary school education, and with respect to the work of the head of the family, most of them (53.8%) are farmers.

From Table 2, there is a difference in the proportion of helminthiasis between before and after the implementation of health education in primary school students, namely, 16.7% and 10%. Statistical analysis showed a significant difference between the proportions of helminthiasis between before and after the provision of health education in elementary school children in North Pontianak (*p* = 0.046).

In Table 3, it can be seen that there is a tendency for school students who have poor hygiene and healthy behavior (31%) to be more infected with helminthiasis than those who have good hygiene and healthy behavior (3.2%). The results of the statistical tests showed that there was a significant relationship between clean and healthy habits with helminthiasis in elementary school children in North Pontianak (*p* = 0.005).

In Table 4, it can be seen that there was a tendency for respondents who were infected with helminthiasis to have anemia (90%), greater than those without worms (48%). Statistical analysis showed a significant association between helminthiasis and the incidence of anemia in primary school children in North Pontianak (*p* = 0.017).

From Table 5, it can be seen that there is a tendency for respondents infected with helminthiasis to have learning achievement below the mean (90%) greater than those who are not infected with helminthiasis (48%). From the results of statistical tests, there was a significant relationship between the incidence of helminthiasis and the learning achievement of elementary school children in North Pontianak (*p* = 0.017).

From Table 6, it can be seen that there is a tendency for respondents who have anemia to have less learning achievement (63.6%) greater than those who are not anemic (44.4%). The results of the chi-square statistical analysis showed that there was a significant relationship between anemia and the learning achievement of elementary school children in North Pontianak (*p* = 0.005).

## 4. Discussion

### 4.1. Worms in Elementary School Children

The proportion of helminthiasis in elementary school children in North Pontianak Subdistrict was 16.7%. This result is lower than the results of an examination of elementary school children in Indonesia with 25% proportion of helminthiasis. Also, our result is low when compared with the results of research in elementary school children in other places, for example, 25% in Semarang and 34.5% in North Sumatra [6, 7]. The low number of helminthiasis in elementary school children in North Pontianak is due to the existence of health care programs through school health efforts. The health services include providing worm medicine and hand washing facilities from the community health center of the Pontianak City Government [4, 17, 18].

The impact of helminthiasis on elementary school children is very serious in terms of both health status and learning achievement [5, 9]. This can be seen from the results of research in Pontianak showing a tendency for health problems and a decrease in learning achievement. Table 1 shows that the majority of respondents (elementary school children) in North Pontianak were anemic by 55% and learning achievement qualifications were below the average of 55%. Children who at the time of school are in the anemic condition usually tend to be lousy or physically unfit and get easily drowsy and tired. As a result of conditions like this, children are lazy to take lessons and concentration and passion for learning decrease [14, 15]. These conditions result in decreased learning achievement so that learning outcomes are below the average class.

### 4.2. Health Education, Clean Behavior, and Worm Infestation

The proportion of helminthiasis in schoolchildren is higher before health education is conducted compared to after the implementation of health education. Health education is carried out to improve the understanding and improvement of knowledge and attitudes and behaviors of a clean and healthy life [4, 17]. Interventions with health education are intended to provide motivation to elementary school children to improve clean and healthy living behaviors so that they can prevent helminthic disorders through the use of environmental health facilities at school and at home. Health education interventions are aimed at improving clean and healthy attitudes and behavior and are useful for increasing community participation through clean and healthy living behaviors to prevent the occurrence of helminthiasis [18, 19]. In addition, health education interventions can be carried out with the aim of improving medical behavior such as going to health services (community health centers) for treatment. In addition, it can also be used to improve clean and healthy lifestyle, such as the habit of washing hands after using the toilet or before eating [20].

Thus, the provision of health education is expected to reduce the incidence of helminthiasis. For this reason, it is recommended that in addition to providing treatment for schoolchildren who are detected with worms, prevention should be also done through regular and routine health counseling for schoolchildren, especially elementary school children. By providing health education, a clean and healthy life behavior will increase, resulting in excellent health conditions, good academic performance, and a clean and healthy environment [21]. The better the behavior of a clean and healthy life, the smaller the occurrence of helminthiasis. For this reason, health education efforts to improve healthy living behavior, especially for schoolchildren, need to be done [21, 22], for example, the habit of cleaning hands and nails, defecating using latrines, and washing hands before eating.

Efforts to improve clean and healthy living behavior can be done through an intervention approach to the efforts of local health services (community health centers) and physical environment control [18, 19]. Health service interventions by community health centers in schools should make students and parents aware of the importance of health care facilities, especially for helminthiasis control. Community behavioral or habitual interventions are intended to increase people's awareness and habits of the importance of clean and healthy lifestyle, while controlling the physical environment is done to provide awareness of the importance of a healthy environment, such as the habit of defecating into healthy latrines. This is expected to be useful to prevent the occurrence of illness and transmission of diseases, especially helminthiasis in elementary school children [21, 22].

### 4.3. Helminthiasis, Anemia, and Student Learning Achievements

Helminthiasis experienced by children especially in elementary school-age children will have a serious influence on their health status and also their learning achievement [5, 9]. Schoolchildren with helminthiasis are anemic by 90%, greater than those without worms. Anemic children will easily get tired and sleepy, and their learning passion will diminish [10]. When schoolchildren are lazy and less passionate about learning, consequently their learning achievements decline [23].

Children who are infected with helminthiasis in the long term will experience a shortage of nutrient intake because the nutrients needed by the body are absorbed by the worms so that children experience anemia. Worm infections can cause chronic bleeding by sticking to the intestinal wall and eating tissues and blood. Blood loss occurs because of blood being sucked by worms and because of bleeding and damaged mucosa, such as Ancylostoma Duodenal which causes blood loss of 0.14-0.4 ml and Necator Americans can reduce blood from 0.01 to 0.4 ml [24]. 1 ml of blood loss will result in iron loss of 0.5 mg, resulting in blood loss of 3-4 ml/day (1.5-2 mg iron), and can result in a negative balance of iron.

Blood loss in schoolchildren is caused by helminth infestations, which attack the proximal small intestine and suck blood from the intestinal submucous veins [23, 24]. Children with anemia have a lower index of psychomotor development than healthy children [25]. Children with low Hb (anemia) have a low learning ability, and their school performance is also lower than that of normal children [26].

## 5. Conclusion

Most students infected with helminthiasis experience anemia and experience a decrease in learning achievement. There are differences in the proportion of helminthiasis between before and after public health counseling. There is a significant relationship between PHBS and helminthiasis. There is a relationship between helminthiasis and anemia and between anemia and learning achievement in children, and there is a significant relationship between helminthiasis and learning achievement in children, especially primary school children.

It is recommended for public health centers to prevent helminthiasis in primary school-age children, in addition to providing medical services such as worm medicine. Besides that, it is important to do health education about the impact of helminthiasis on health status and learning achievement. The school is expected to provide understanding about the effects of helminthiasis on health and sanitation facilities and hand washing facilities in schools. For parents, they are expected to play an active role in reminding clean and healthy lifestyle habits at home such as encouraging children to wear footwear when doing activities outside the home, washing hands before eating, and washing hands after using the toilet.

## Figures and Tables

**Table 1:** Characteristics of respondents.

Variables	*N* = 60	Percentage (%)
Gender		
Male	38	63.3
Female	22	36.7
Helminthiasis		
Positive	10	16.7
Negative	50	83.3
Anemia status		
Yes	33	55.0
No	27	45.0
Learning achievement		
<Mean	33	55.0
≥Mean	27	45.0
Clean and healthy living behavior (PHBS)		
Less	29	48.3
Good	31	51.7
Mother's education level		
Primary	30	50.0
Secondary	20	33.3
Tertiary	10	16.7
Father's education level		
Primary	25	41.7
Secondary	20	33.3
Tertiary	15	25.0
Family status		
Single parents	10	16.7
Complete parents	50	83.3
The job of the head of the family		
Farmer	35	58.3
Private	15	25.0
Government employees	10	16.7

**Table 2:** Health education and helminthiasis.

Health education	*N*	Proportion (%)	*p* ^a^
Before	60	16.7	0.046^∗^
After	60	10.0	

**Table 3:** Clean and healthy living behavior and helminthiasis.

Clean and healthy living behavior	Helminthiasis				Total		*p* ^a^
	Positive		Negative				
	*n*	%	*n*	%	*N*	%	
Less	9	31	20	69	29	100	0.005^∗^
Good	1	3.2	30	96.8	31	100	

**Table 4:** Helminthiasis and anemia.

Helminthiasis	Anemia				Total		*p* ^a^
	Yes		No				
	*n*	%	*n*	%	*N*	%	
Positive	9	90	1	10	10	100	0.017^∗^
Negative	24	48	26	52	50	100	

^a^Chi-square.

**Table 5:** Helminthiasis and achievement of learning.

Helminthiasis	Achievement of learning				Total		*p* ^a^
	<Average		≥Average				
	*n*	%	*n*	%	*N*	%	
Positive	9	90	1	10	10	100	0.017^∗^
Negative	24	48	6	52	50	100	

**Table 6:** Anemia and achievement of learning.

Anemia	Achievement of learning				Total		*p* ^a^
	<Average		≥Average				
	*n*	%	*n*	%	*N*	%	
Yes	21	63.6	12	36.4	33	100	0.005^∗^
No	12	44.4	15	56.6	27	100	

## Data Availability

The research data used to support the findings of this study are available from the corresponding author upon request.
